# Pocket Doppler and vascular laboratory equipment yield comparable results for ankle brachial index measurement

**DOI:** 10.1186/1471-2261-8-26

**Published:** 2008-10-07

**Authors:** Saskia PA Nicolaï, Lotte M Kruidenier, Ellen V Rouwet, Liliane Wetzels-Gulpers, Constantijn AM Rozeman, Martin H Prins, Joep AW Teijink

**Affiliations:** 1 Department of Surgery, Atrium medical center, Henri Dunantstraat 5, 6419 PC Heerlen, the Netherlands; 2 Department of Clinical Neurophysiology/non-invasive vascular lab, Atrium medical center, Henri Dunantstraat 5, 6419 PC Heerlen, the Netherlands; 3 Department of Epidemiology, Maastricht University/KEMTA, PO Box 616, 6200 MD Maastricht, the Netherlands

## Abstract

**Background:**

The ankle brachial index (ABI) is a well-established tool for screening and diagnosis of peripheral arterial disease (PAD). In this study we assessed the validity of ABI determination using a pocket Doppler device compared with automatic vascular laboratory measurement in patients suspected of PAD.

**Methods:**

Consecutive patients with symptoms of PAD referred for ABI measurement between December 2006 and August 2007 were included. Resting ABI was determined with a pocket Doppler, followed by ABI measurement with automatic vascular laboratory equipment, performed by an experienced vascular technician. The leg with the lowest ABI was used for analysis.

**Results:**

From 99 patients the mean resting ABI was 0.80 measured with the pocket Doppler and 0.85 measured with vascular laboratory equipment. A Bland-Altman plot demonstrated great correspondence between the two methods. The mean difference between the two methods was 0.05 (P < .001). Multivariate linear regression analysis showed no dependency of the difference on either the average measured ABI or affected or unaffected leg.

**Conclusion:**

Since the small, albeit statistically significant, difference between the two methods is not clinically relevant, our study demonstrates that ABI measurements with pocket Doppler and vascular laboratory equipment yield comparable results and can replace each other. Results support the use of the pocket Doppler for screening of PAD, allowing initiation of cardiovascular risk factor management in primary care, provided that the equipment operator is experienced.

## Background

The ankle brachial index (ABI) is useful in the diagnosis of peripheral arterial disease (PAD). With a sensitivity and specificity of 90% and 98%, respectively, ABI is especially helpful in establishing lower extremity PAD [1,2]. The ABI has become increasingly important as a screening tool for identification of patients with asymptomatic PAD [3], which is an independent marker for adverse cardiovascular outcome [4]. A patient with a low ABI has a 5.5-fold increased risk of cardiovascular death and a 2.5-fold higher risk of coronary artery disease and of stroke [5]. Current guidelines recommend initiation of secondary prevention measures in all patients with a screening ABI value < 0.9 and treatment of atherosclerosis risk factors [3]. Given the importance of the ABI as a predictor of cardiovascular disease and mortality [6], accurate determination of the ABI is crucial.

Equipment used to measure arm and ankle pressures differs between the primary care setting, outpatient clinics, and the vascular laboratory setting. Arm and ankle pressures in primary care and in outpatient clinics are usually measured with a pocket Doppler device. In vascular laboratory settings, these measurements are performed with automatic vascular laboratory equipment. Although the pocket Doppler method is widely used, comparisons of this method with vascular laboratory equipment have been limited. A recent study compared the ABI measurements of 30 patients with both types of equipment [7]. The pocket Doppler measurement was performed by a nurse, while the measurement in the vascular laboratory was performed by a vascular technologist, and the two health care providers had different levels of expertise in performing ABI measurements. The aim of this study was to compare pocket Doppler ABI measurements with automatic measurements performed in a vascular laboratory, with the procedures being performed by observers with equal levels of expertise and to determine if there are intrinsic differences in the results obtained with these two devices.

## Methods

Ninety nine consecutive patients suspected of PAD who had been referred to the vascular laboratory of our hospital for an ABI measurement between December 2006 and August 2007 were included in this study. Informed consent was obtained and the study was approved by the medical ethical committee of the Atrium medical center Parkstad.

For valid comparisons of ABI measurements performed by pocket Doppler and with laboratory equipment, both measurements were conducted on the same day in the vascular laboratory. For both methods, brachial pressures were measured bilaterally, and were repeated if the difference was > 10 mm Hg between the two arms. Ankle pressures were determined with cuffs placed proximal to the malleoli. Following a 15 minute resting period, systolic blood pressures (SBP) in the brachial, dorsal pedal, and posterior tibial arteries were determined in a supine position with a pocket Doppler device (Doppler MD2, Huntleigh Healthcare, Cardiff, United Kingdom) by a trained vascular laboratory professional. Brachial and ankle pressures were measured with a sphygmomanometer cuff which was inflated and deflated manually. The first audible signal of the first ventricular systole was used to identify the SBP at each location. The ABI was calculated by dividing the highest systolic ankle pressure (either posterior tibial or dorsal pedal) in each leg by the highest systolic brachial pressure [8,10]. Then, all measurements were repeated by a second vascular technician, blinded to the previous results, using laboratory equipment (VasoGuard System XP84 (1999), Scimed, Bristol, United Kingdom). Sphygmomanometer cuffs used to measure brachial and ankle pressures inflated and deflated automatically by pressing a button. The SBP cut-off points of all arteries were defined as the systolic upstroke of the first arterial waveform. At the first characteristic arterial sound and at the simultaneous appearance of the first arterial waveform, the monitor screen was frozen, and the SBP cut-off point was defined by precise retrospective positioning of an adjustable marker line (Table 1).

**Table 1:** Comparison of pocket Doppler and vascular laboratory measurement of brachial and ankle blood pressures

	**Pocket Doppler**	**Vascular laboratory equipment**
Probe	8 MHz	8 MHz
Cuff inflation/deflation	Manually	Automatically
SBP cut-off point	• Audible signal	• Visual and audible signal
	• View the manometer at the time of the first sound	• Adjustable line placed on the monitor
	• Rapid response with immediate determination of the cut-off point	• Line is precisely positioned retrospectively

### Statistic evaluation

ABI measurements for each leg of the same patient are probably correlated since atherosclerosis is a generalised disease. Therefore, we used the lower ABI of both legs of each patient for analyses. The ABI values obtained from the pocket Doppler and from the vascular laboratory were averaged. The leg affected with PAD was defined as a leg with an ABI < 0.9. Differences between measurements were assessed with a one-sample Student's *t*-test. Multivariate linear regression analysis assessed the dependency of the observed difference between the two measurements and the average measured ABI for the affected and unaffected legs. Due to ethical considerations, intra arterial blood pressures were not performed and a Bland-Altman plot was used to visualise agreement between the two methods [9]. Statistical analysis was performed with SPSS version 14.0 for Windows.

## Results

Characteristics of the study population are presented in Table 2. The mean age of the 99 participating patients was 65.0 years. Characteristics of the study population are presented in table 2. In total, 56 legs were diagnosed with PAD, (23 right legs and 33 left legs). The mean ABI of the 99 patients was 0.80 (SD 0.25) as measured with the pocket Doppler and 0.85 (SD 0.25) as measured with vascular laboratory equipment. Subtraction of the pocket Doppler result from the automatic vascular laboratory equipment result yielded a mean difference of 0.05 (SD 0.09), a value that was statistically significant (*P *< .001). Multivariate linear regression analyses showed no dependency of the difference on the average measured ABI (P = .187) or whether the measurements were performed on affected or unaffected legs (P = .235).

**Table 2:** Clinical characteristics of the study population

	Analysed population n = 99
Men – n (%)	60 (60.6)
Age (years) – mean (SD)	65.0 (12.2)
BMI – mean (SD)	26.6 (3.9)
Hypertension – n (%)	72 (72.7)
Diabetes Mellitus – n (%)	27 (27.3)

**Figure 1:**
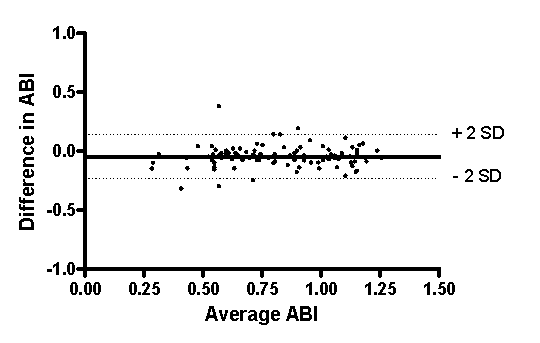
**Bland-Altman plot**. Bland-Altman plot: The average ABI of pocket Doppler and vascular laboratory equipment plotted against the difference in ABI of both measurements. (n = 99).

The two methods were compared by a Bland-Altman plot (Figure 1) which depicts the average ABI as determined by the two measurements across the difference between the pocket Doppler and the automatic ABI measurement for each leg. The Bland-Altman analysis confirmed that the data obtained by the two procedures was virtually the same.

## Discussion

This study shows that the ABI values determined by a simple pocket Doppler device and by automatic vascular laboratory equipment are interchangeable. In view of the importance of the ABI in detecting patients with atherosclerosis, our study supports the use of the easily accessible and applicable Doppler device for the screening and diagnosis of PAD, thus permitting the initiation of cardiovascular risk factor management in the primary care practice.

The good clinical interchangeability between ABI assessment with pocket Doppler and automatic vascular laboratory equipment was elegantly demonstrated by a Bland-Altman plot. However, ABI, as measured by pocket Doppler, tended to be consistently lower, independent of the average of the measured ABI and whether measurements were obtained from the affected or the unaffected leg. Although the minor, albeit statistically significant, difference in ABI of 0.05 is not considered to be clinically relevant [10], it may be important in the epidemiological context. In larger studies, ABI values close to the cut off point of 0.9 could influence the reported prevalence of PAD [11], and affect the determinations of sensitivity and specificity of the ABI measurement for the identification of high risk patients. An additional study on a larger population is required to resolve this problem.

The small difference in ABI between both methods may relate to the method of determination of SBP cut-off points and cuff in- and deflation. With the pocket Doppler, SBP is recorded from the sphygmomanometer simultaneously with the first audible signal, which can be influenced by human auditory limitations as well as by a slow response to the rapidly occurring audible signal. Most laboratory equipment automatically visualises the Doppler signal output with spectral analysis and displays the entire frequency and amplitude of the Doppler signal on the monitor [12]. The screen is frozen as the first arterial waveform is displayed and is accompanied by the audible signal. Subsequently, an adjustable line is placed precisely at the systolic upstroke of the first arterial waveform. Other artefacts including slight movements of the hand holding the Doppler device during manual inflation and deflation, alterations in the position or angle of the device, and variations in the amount of pressure can affect the quality of the Doppler signal, and consequently the SBP measurement [13].

In general, screening for PAD by ABI and thus, screening for atherosclerosis in peripheral arteries of the leg as a reflection of generalised atherosclerosis is highly encouraged. However, we suggest that clinical judgement must be used in the interpretation of ABI values determined by pocket Doppler. Diabetes or longstanding renal failure medial calcinosis could lead to calcified arteries which may be inadequately compressed by the sphygmomanometer cuff, leading to falsely elevated ankle pressures. Referral to a vascular laboratory for the measurement of systolic toe pressures or additional vascular imaging is essential for adequate determination of the vascular status of these patients [10].

Furthermore, an experienced operator is mandatory for accurate determination of the ABI, as previously indicated by the positive influence of experience and training on the reproducibility of the ABI measurement [14,15]. Ray *et al. *demonstrated that inexperienced doctors performed ABI measurements less reliably than their trained counterparts [16]. In the present study, all vascular technicians were trained and experienced. Since the pocket Doppler method is highly operator dependent, it is particularly important that medical personnel is adequately trained in the acquisition of data using the pocket Doppler device so that ABI assessment can be widely applicable in primary care practice. However, the advised methods reported in the literature vary for the performance and the calculation of ABI measurement [10,12,17]. Ideally, guidelines consistent with the method of measurement and calculation of the ABI should be established. Indeed, this instruction could be the basis for structured training programmes for medical personnel to develop this expertise. The present study shows that the pocket Doppler and automatic vascular laboratory equipment measurements of the ABI are interchangeable in patients suspected of PAD. The validity of pocket Doppler ABI measurement in a screening setting could be a subject of future studies.

## Conclusion

Pocket Doppler assessment was demonstrated to be a practical tool for reliable and quick evaluation of the vascular status of a patient. This provides a useful tool for the investigation of patients with lower limb pain, and enables the targeted referral of patients with symptomatic PAD to the vascular specialist. Even more importantly, it introduces the opportunity for atherosclerosis screening and cardiovascular risk management in asymptomatic patients to reduce cardiovascular morbidity and mortality.

## Competing interests

The authors declare that they have no competing interests.

## Authors' contributions

SN carried out the statistical analysis and was responsible for writing the drafts of the manuscript. LK contributed to the preparation of the manuscript and the statistical analysis.  EV reviewed the manuscript critically. LW carried out the ABI measurements and contributed to the preparation of the manuscript. CR participated in development of the concept and design of the study and reviewed the manuscript. MH contributed to the concept and design of the study and to the statistical analysis. JT participated in the conception and design of the study and critically reviewed the manuscript.

## Pre-publication history

The pre-publication history for this paper can be accessed here:


